# Ultrastructural regenerating features of nasal mucosa following microdebrider-assisted turbinoplasty are related to clinical recovery

**DOI:** 10.1186/s12967-016-0931-8

**Published:** 2016-06-08

**Authors:** Giampiero Neri, Fiorella Cazzato, Valentina Mastronardi, Mara Pugliese, Maria Antonietta Centurione, Roberta Di Pietro, Lucia Centurione

**Affiliations:** Department of Neuroscience and Imaging, Faculty of Medicine, University “G. d’Annunzio”, Chieti-Pescara, Italy; Institute of Molecular Genetics, National Research Council, Pavia, Section of Chieti Italy; Department of Medicine and Aging Sciences, Section of Human Morphology, University “G. d’Annunzio”, Chieti-Pescara, Italy

**Keywords:** Nasal mucosa, Rhinitis, Turbinoplasty, Microdebrider, Stem cells, Electron microscopy

## Abstract

**Background:**

The nasal mucosa plays a key role in conditioning the inhaled air and in regulating the immune response. These functions led many authors to recommend mucosal sparing techniques for the surgical management of inferior turbinate hypertrophy. However, the histological modifications of chronic diseases retain the inflammatory activity and prevent the nasal physiology restoration. It has been proved that the basal cells of the nasal mucosa are able to proliferate and to repair after cold-knife incision. The aim of this study was to demonstrate that the healing process after removal of the inferior turbinate mucosa with cold techniques results in a complete structural restoration.

**Methods:**

A prospective study was performed in 18 patients who underwent Microdebrider inferior turbinoplasty (cold technique). Subjective and objective improvement of nasal patency was evaluated with visual analogue scale, rhinomanometry, videoendoscopy and mucociliary transport test. Pre- and post-operative biopsy specimens were taken from 7 patients to evaluate the healing process. Two samples were taken from two healthy patients as control. The specimens were processed for transmission electron microscopy analysis.

**Results:**

Videoendoscopy showed reduction of lower turbinate after surgery. Nasal patency augmented and no adverse consequences were observed. After 4 months the nasal mucosa showed normal appearance, with restoration of the pseudostratified ciliated pattern, intercellular connections and normal cellular morphology. Fibrosis and submucosal edema disappeared. At longer time after operation (4 years) clinical improvement was confirmed.

**Conclusions:**

The total removal of the nasal mucosa with cold techniques results in a complete restoration of the normal structure and permanent resolution of the chronic inflammation typical of hypertrophic rhinopathy.

## Background

Chronic nasal obstruction is a condition frequently encountered in rhinological practice. It can interfere with social and business activities and negatively affects the quality of life [1, 2].

The most common cause of this disease is the chronic hypertrophic rhinitis with a remarkable reduction of nasal patency [1].This condition can be observed mostly in persistent rhinitis, septum deviation [3] and other cases [4].

An increase in turbinate size is associated with histological mucosal and submucosal changes. [1, 5, 6] The damage of the epithelial barrier leads to an increase in vascular and secretory reflexes and hyper-responsiveness of sensory nerves [1, 4, 7, 8].

The patients usually complaints of sneezing, nasal pruritus, rhinorrhea, blocked nasal passages, post nasal drip, congestive fullness at the root of the nose, and frontal headache. Other symptoms experienced include dryness of the throat and pharynx, ear pain, snoring, sleep disturbance, allodynia, hyperalgesia and hyposmia [1, 9, 10].

The diagnosis is based mainly on the patient’s history, inspection of the external and inner nose, endoscopy of the nasal cavities, and paranasal sinuses, allergy testing, rhinomanometry and mucociliary transport test [11].

When medical therapyfails [9, 10, 12, 13], surgical reduction of the inferior turbinatesis suggested. Conventional surgical options are total or partial turbinectomy [14] but there is no clear consensus in the literature indicating the “gold standard” technique for turbinate reduction [1, 15]. The main aim of turbinate surgery is the restoration of the nasal respiratory volume to ensure the humidification and the purification of the air, maintaining nasal function and minimizing complications [1, 11, 16–18]. It is common opinion that the preservation of mucosal layer is mandatory to achieve this goal. Nevertheless saving an altered mucous tissue may support the maintenance of a chronic inflammatory process resulting in recidivism [11].

In the past years several studies have been performed on the ultrastructural changes of the nasal mucosa after different surgery techniques but all the methods consisted in a reduction of the stromal tissue of the lamina propria with preservation of the mucosal layer [5, 7, 19].In a recent study, carried out by our research group on patients subjected to Microdebrider-assisted turbinoplasty, we were able to identify the healing of the mucosal surface by means of scanning electron mycroscopy [20] but we had no information about the ultrastructural characteristics of all the mucosal layers.

Thus, the aim of this study was to demonstrate, through transmission electron microscopy analysis, that the total removal of the nasal mucosa with the microdebrider, a particular type of cold technique, results in a complete ultrastructural restoration of the healthy tissue.

We also evaluated objective and subjective outcomes in order to demonstrate the importance of total removal of the damaged tissues for a complete restoration of a healthy nasal mucosa.

## Methods

A prospective study was performed in 18 patients (12 Males and 6 Females), ranging in age from 15 to 79 years (mean age 36.77 ± 16.46 years), with prolonged nasal obstruction due to inferior turbinates hypertrophy. All patients underwent endoscopic Microdebrider-assisted inferior turbinoplasty under local anesthesia at the outpatient clinic of the Department of Otolaryngology, SS Annunziata Hospital, Chieti, Italy.

The diagnosis was based on history, clinical examination, nasal endoscopy, rhinomanometry, and functional tests.

All participants had to satisfy the following requirements:

- persistent symptoms and signs of severe nasal obstruction related to hypertrophic turbinates;
- poor response to medical treatment;
- presence of complications of nasal blockage (otitis, pharyngitis, tracheitis, OSAS);
- inefficacy of immunotherapy in patients with allergic rhinitis.

Cases with septal nose deviations, sinonasal polyposis or tumors, history of a sinus or nasal surgery, adenotonsillar hypertrophy, ciliary diskinesia caused by genetic diseases, previous head and neck radiotherapy were excluded from the study. Two biopsies of normal mucosa were taken from 2 healthy patients considered as control. Informed consent was obtained from all the patients who received detailed information about the study adhering to the Declaration of Helsinki and to the ICH-GCP, GU 184/2003.

### Surgical technique

The Xoomed Power System 2000 Microdebrider was used to carry out the procedure under 0° endoscopic guidance. The inferior and medial portions of the inferior turbinate above the periosteum were shaved using the Microdebrider in the oscillating mode. Hemostasis was achieved by nasal packing of a biocompatible synthetic polymer of esterified polyvinyl derivates with hyaluronic acid of 8 or 10 cm in length for 72 h. After pack removal the patients began a local instillation of nose drops containing vitamin A and Vaseline oil for at least 1 month.

### Subjective and objective evaluations

Prior to surgery and after a follow up period of 4 months, participants underwent a battery of subjective and objective evaluations. Each patient was subjected to a complete ENT clinical examination. Nasal endoscopy was performed to evaluate the extent of the enlargement of the concha. The subjective severity of nasal obstruction was assessed by using a standard 10 cm Visual Analogue Scale. Mucociliary transport time was performed using the saccharin transit time [21].

Anterior Active Rhinomanometry was performed using ATMOS 300 rhinomanometer. The measurements were obtained at a sample pressure point of 150 Pa before and after application of 0.1 % naphazoline hydrochloride.

Digital images were acquired by means of a ZEISS Axioskop light microscope equipped with a Coolsnap video camera and MetaMorph software.

## Results and discussion

All patients were satisfied with the result of the procedure and experienced a great improvement of symptoms and quality of life. None of the patients had any postoperative bleeding after removal of nasal packing. Complications such as crusts, bleeding, foul odor, atrophic changes or recidivism were not observed during the 4 months follow up. At 4 years after surgery, clinical assessment confirmed the restoration of nasal physiology. All data are shown in Table 1.

**Table 1:** Preoperative and postoperative clinical assessment values

Patient	Age	Gender	VAS1	VAS2	VAS3	VAS4	TMC1	TMC2	TMC3	TMC4	RMM1	RMM2	RMM3	RMM4
D.P.A	44	M	10	6	2	1	16	10	7	5	1.20	0.20	0.33	0.39
R.P	60	M	9	3	1	1	15	11	8	6	1.80	0.28	0.57	0.79
D.P.F	15	M	9	2	1	1	15	13	6	4	1.60	0.30	0.45	0.37
L.A	35	F	8	5	3	2	14	9	5	3	1.38	0.31	0.27	0.32
T.S	30	M	10	5	2	1	17	13	5	5	1.40	0.48	0.43	0.37
D.L.L	33	M	9	6	1	1	19	9	6	5	1.14	0.27	0.35	0.33
D.A.M	57	F	10	7	2	1	18	8	8	5	1.36	0.83	0.67	0.91
F.R	32	M	9	5	1	1	13	12	6	3	1.30	0.31	0.45	0.55
R.M.R	32	F	7	6	2	1	12	9	4	2	1.20	0.22	0.54	0.34
D.S.A	26	M	7	5	1	2	15	10	5	4	1.40	0.23	0.26	0.44
M.G	17	F	8	4	1	1	16	9	4	3	1.20	0.13	0.33	0.55
C.S	25	M	9	6	1	1	16	9	4	4	1.09	0.11	0.67	0.72
M.A	41	M	8	4	1	1	15	11	7	8	1.11	0.36	0.44	0.56
B.V	28	M	8	5	1	2	16	9	9	7	1.50	0.58	0.66	0.73
N.A	68	M	7	3	1	1	15	10	8	7	1.02	0.32	0.57	0.88
B.R	66	M	8	4	1	1	16	11	7	5	1.08	0.22	0.55	0.44
D.L.S	53	F	8	4	1	1	12	11	3	2	1.10	0.12	0.35	0.49
C.R	F	F	9	6	1	1	19	12	7	6	0.80	0.31	0.24	0.37
Mean value			8.5	4.61	1.38	1.16	15.5	10.3	6.05	4.66	1.26	0.31	0.45	0.53
Standard deviation			0.97	0.97	0.76	0.37	2.25	1.61	1.65	1.67	0.86	0.15	0.14	0.19

VAS-Visual analogue Scale (10 indicates severe nasal obstruction, 1 indicates absence of nasal obstruction). TMC-Mucociliary transport time (Normal TMC ≤ 13 ± 3 min). RMM: Rhinomanometry (range 0.30–0.60 Pa/cm^3^/s). VAS1, TMC1, RMM1: preoperative; VAS2, TMC2, RMM2: 1 week; VAS3, TMC3, RMM3: 4 months; VAS4, TMC4, RMM4: 4 years

### Improvement of symptoms

VAS scores showed a significant difference when compared with the scores before operation. The mean preoperative VAS (VAS1) for nasal obstruction was 8.5 ± 0.97 and it decreased to 4.61 ± 0.97 at 7 days (VAS2) in the 61.1 % of volunteers, and to 1.38 ± 0.76 in 88.8 % of the patients at 4 months after surgery (VAS3). At longer times after surgery (4 years) VAS decreased in 100 % of volunteers to 1.16 ± 0.37 (VAS4).

### Improvement of nasal patency

Endoscopy confirmed the improvement of nasal patency. The volume of nasal cavities increased and the conchal form was conserved. The mucosa appeared healed, pink colored, without crusts, synechiae and submucosal edema.

Rhinomanometric assessment revealed a reduction of nasal resistance. Before surgery the mean value, calculated at 150 Pa of pressure, was 1.26 ± 0.86 Pa/cm^3^/s (RMM1). After 7 days it decreased to 0.31 ± 0.15 Pa/cm^3^/s (RMM2). At 4 months was 0.45 ± 0.14 Pa/cm^3^/s (RMM3) and after decongestion test was 0.11 Pa/cm^3^/s. At 4 years measured 0.53 ± 0.19 Pa/cm^3^/s (RMM4).

Mucociliary transport time passed from a mean value of 15.5 ± 2.25 min preoperatively (TMC1) to 10.3 ± 1.61 min after 7 days (TMC2). At 4 months passed to 6.05 ± 1.65 min (TMC3). At 4 years decreased to 4.66 ± 1.67 min (TMC4).

## Structural and ultrastructural analysis

### Preoperative mucosa

**Fig. 1:**
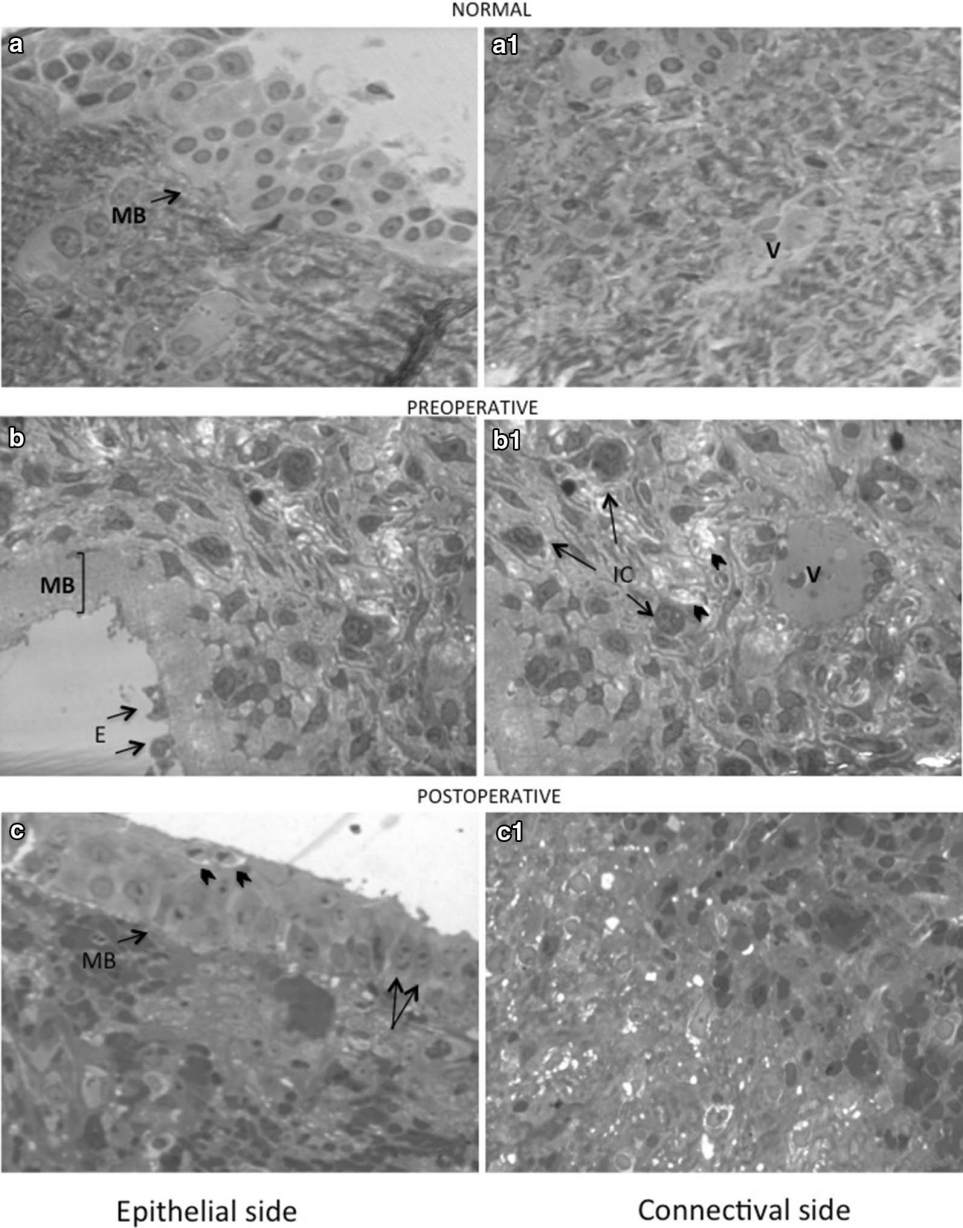
Structural patterns in control, preoperative and postoperative specimens of nasal mucosa (40×). **a** Normal ciliated pseudostratified epithelium on thin basement membrane (BM); the normal organization of the extracellular matrix with small blood vessels (V) is visible in the connective tissue underneath (**a**_**1**_). **b** Preoperative mucosa showing the focal thickening of the basement membrane (BM) completely exposed to the external environment; epithelium is partially or completely missing with small residues of epithelial cells (EEC) flaking off. In the connective tissue (**b**_**1**_) numerous inflammatory cells (IC) are immersed in an abundant amorphic substance (*arrowheads*) with few collagen fibers. **c**: Re-epithelisation of postoperative mucosa with the initial organization of the pseudostratified columnar epithelium (*arrows*) above a normal thickness basement membrane (BM). Some apoptotic cells are visible at the surface (*arrowheads*). Connectival side shows an evident recovery (**c**_**1**_) with reduction of the inflammatory infiltrate and abundant amorphic substance

Light microscopy analysis performed on semithin sections from preoperative specimens of all the subjects displayed the characteristics of a severe mucosal damage in comparison with healthy control samples (Fig. 1a–a_1_). The normal pseudostratified columnar ciliated epithelium frequently degenerated to flattened or squamous stratified epithelium with marked reduction of goblet cells number. In some regions the epithelial layer was completely missing and showed small residues of epithelial cells flaking off, degenerating ciliated cells deprived of cilia and microvilli and therefore alteration of the cell contour. Beneath the epithelium, reduced in thickness or completely absent, the basement membrane appeared to be focally very thickened or, more rarely, rather thinned (varying from a maximum of 23.87 μm to a minimum of 4.29 μm). The thickened portion of the basement membrane appeared to be related to the complete absence of the epithelium, remaining fully exposed to the external environment. Consequently, the underlying connective tissue was infiltrated by numerous inflammatory cells of different types immersed in an extracellular matrix, wherein the amorphic substance was abundant and the collagen fibers very rare (Fig. 1b–b_1_).

**Fig. 2:**
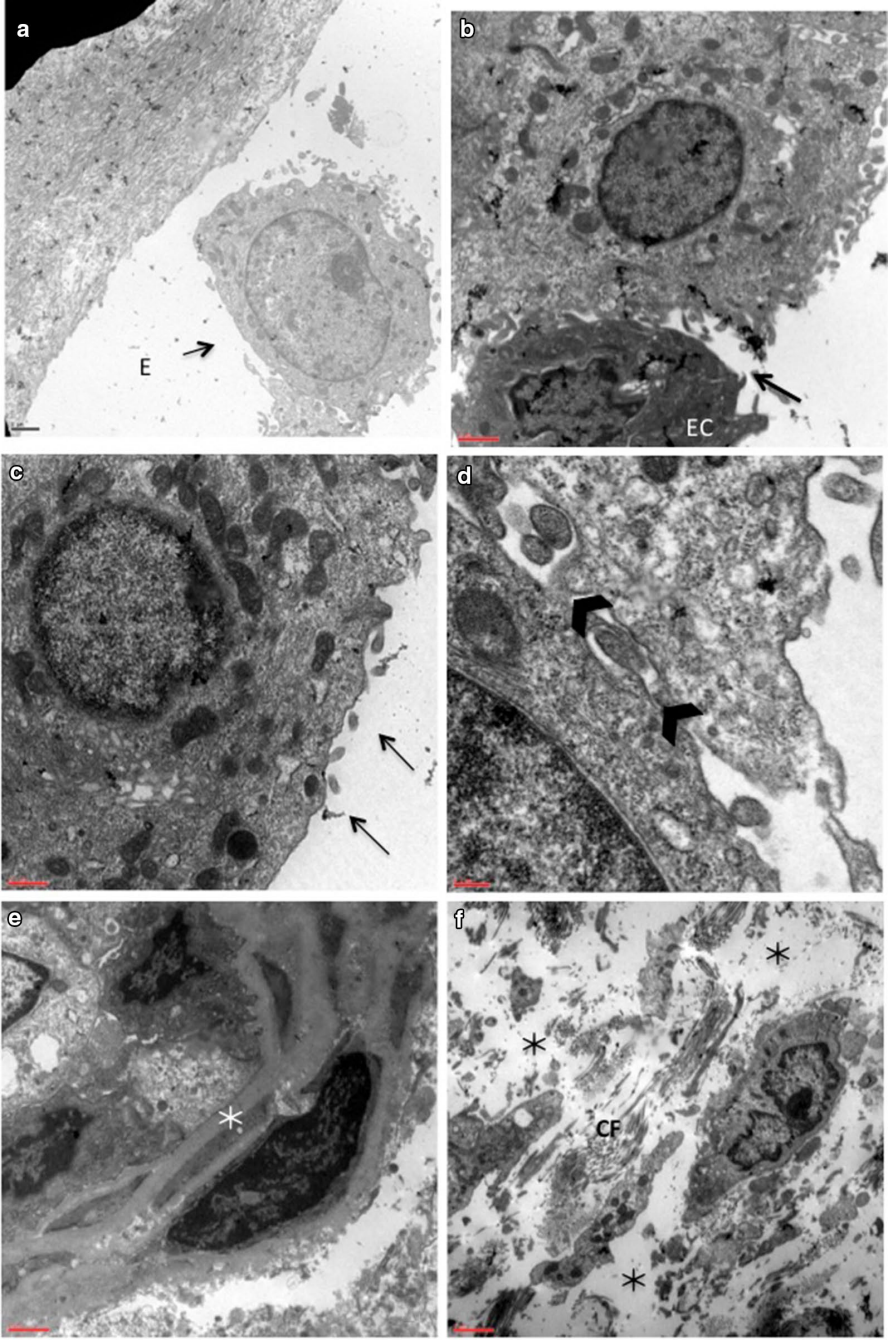
Ultrastructural patterns in preoperative specimens of nasal mucosa. **a** Epithelial cell (EC) flaking off from a thickened basement membrane (BM). *Scale bar* = 1 μm. **b** Dark and shrinking epithelial cell (EC) losing contact with neighbouring cells (*arrow*). *Scale bar* = 1 μm. **c** Epithelial cell flattened surface devoid of surface specializations (*arrows*). *Scale bar* = 0.5 μm. **d** Disrupted structure of intercellular junctions (*arrowheads*). *Scale bar* = 0.5 μm. **e** Thickened basement membrane (*white asterisk*) of blood capillaries. *Scale bar* = 1 μm. **f** Extracellular matrix containing abundant amorphic substance (*black asterisk*) and fragmented collagen fibers (CF). *Scale bar* = 2 μm

At ultrastructural level, all the observations were confirmed: in preoperative mucosa, the partial or complete detachment of epithelial cells (EC) related to degraded and damaged intercellular junctions with linear approach of cell to cell membrane apposition and consequent distension of the intercellular spaces (Fig. 2b, d). Cells exhibited signs of cellular suffering such as pyknotic and indented nuclei with dark cytoplasm, in which are evident ailing organelles: dilated endoplasmic reticulum, abnormal mitochondria, shrunk packed microfilaments and rare ribosomes (Fig. 2b–d). Unusual residuals of cilia and microvilli are present at the epithelial surface (Fig. 2d). Below the epithelium the thickened basement membrane showed an altered and homogeneous structure (Fig. 2a). The extracellular matrix showed macrophages, neutrophils, eosinophils and dendritic cells (Fig. 1b–b_1_, Fig. 2e, f), abundant amorphic substance and few fragmented collagen fibers (Fig. 2f). Neutrophilic granulocytes presented cytoplasmic vacuolization and degranulation. Some macrophages were observed during phagocytosis. The venous sinusoids and capillaries were congested with marked deformity of endothelial cells (Fig. 2e).

### Postoperative mucosa

Postoperative samples, obtained 4 months after surgery, revealed all the signs of the healing process towards normal patterns restoration. In semi-thin sections, in fact, the re-epithelization of the mucosa was evident, showing the initial organization of the pseudostratified columnar epithelium above a normal thickness basement membrane (BM). Some apoptotic cells were also evident at the epithelial surface. Likewise, the connective tissue was devoid of inflammatory infiltrates and displayed a greater amount of collagen fibers (Fig. 1c, c_1_).

**Fig. 3:**
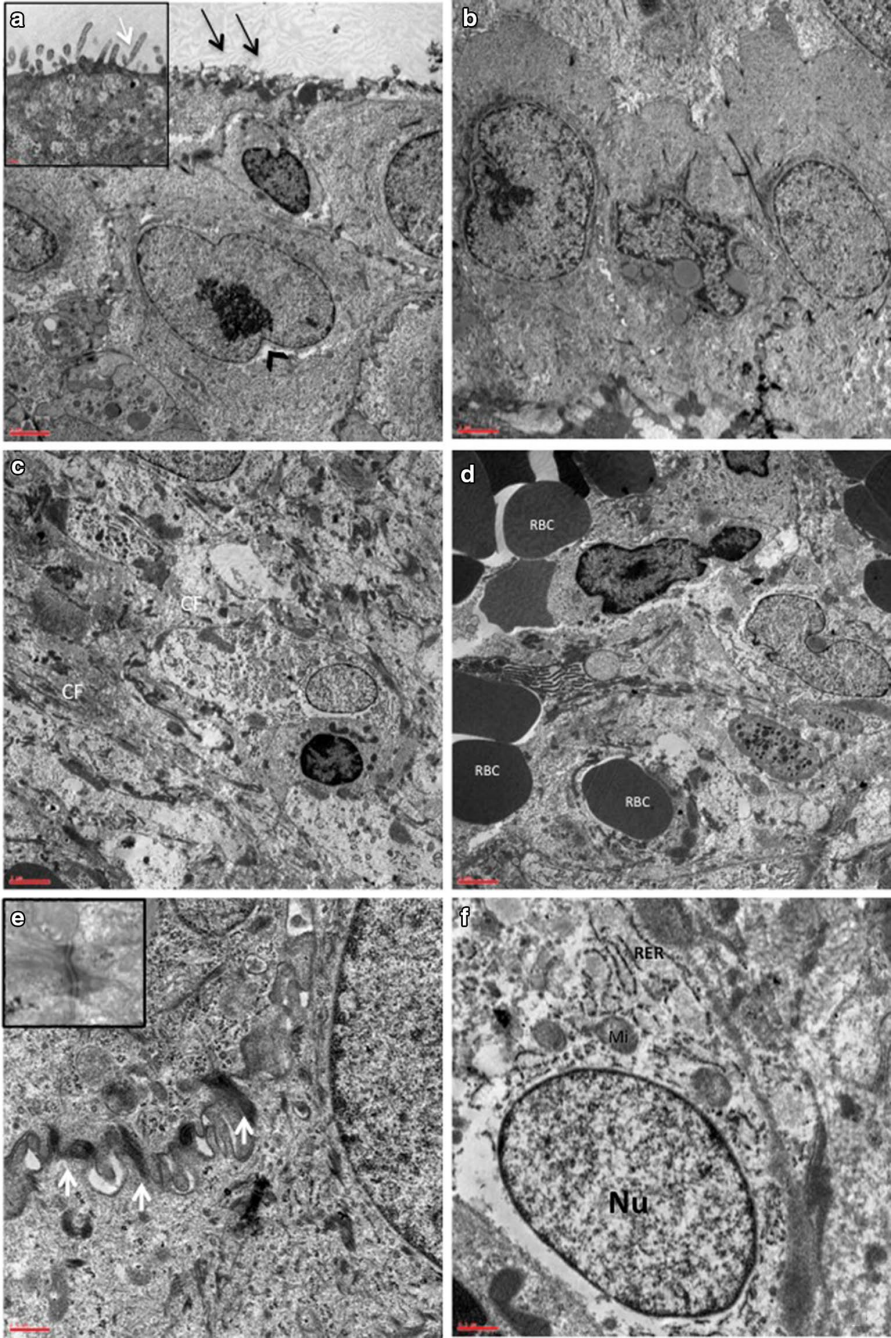
Ultrastructural patterns in postoperative specimens of nasal mucosa. **a** Re-epithelisation with increased cell proliferation (*arrowhead*) and reorganization of surface specializations (*arrows*), showing dichotomous branching of microvilli with fibrillary structure, better visible in the *inset* at higher magnification (*white arrow*). *Scale bar* = 2 μm; *Inset* *Scale bar* = 0.5 μm. **b** Columnar pattern of restored epithelial cells. *Scale bar* = 2 μm. **c** Extracellular matrix showing normal ratio between amorphic substance and collagen fibers (CF). *Scale bar* = 2 μm. **d** Presence of blood vessels containing red blood cells (RBC). *Scale bar* = 2 μm. **e** Reorganization of desmosomes (*inset*) and interdigitated cell junctions. *Scale bar* = 0.5 μm. **f** Euchromatic nucleus (Nu) in a metabolically active cell rich in organelles such as rough endoplasmic reticulum (RER) and mitochondria (Mi), diffuse ribosomes and polyribosomes. *Scale bar* = 0.5 μm

Transmission Electron Microscopy observations of postoperative mucosa samples (Fig. 3) confirmed the presence of a restored epithelium with well-organized ciliated cells, brush cells, goblet cells and basal cells (Fig. 3a). Cells displayed euchromatic nuclei with prominent nucleoli and abundant cytoplasmic organelles such as rough endoplasmic reticulum (RER), mitochondria, ribosomes and polyribosomes (Fig. 3f). They increased proliferation and frequently reorganized in a columnar pseudostratified pattern, anchored each other by interdigitated junctional complexes including tight junctions, desmosomes and gap junctions (Fig. 3e). Regarding cell surface, we observed the reorganization of surface specializations, particularly the dichotomous branching of microvilli with their fibrillary structure, typical of human respiratory nasal mucosa (Fig. 3a). Ultrastructural analysis of connective tissue revealed an extracellular matrix showing a normal ratio between amorphic substance and collagen fibers, typically arranged in bundles (Fig. 3c). As demonstrated in light microscopy, fewer inflammatory cells were visible, in the presence of little blood vessels with normal thickness of the basement membrane (Fig. 3d).

The Microdebrider-assisted turbinoplasty is able to allow the re-epithelization process of nasal mucosa by removing inflamed soft tissue without burning resection margins.

The respiratory mucosa lines the maxillo-turbinal region of the nasal cavities. The pseudostratified columnar epithelium consists of basal cells, intermediate cells, ciliated columnar cells and goblet cells and rests on a basement membrane. The submucosal lamina propria is composed of a loose connective tissue with a rich vascular network, seromucous glands and mesenchymal cells [22–26]. This tissue plays a major role in nasal physiology by conditioning the inhaled air and mediating the immune response [2, 24, 27]. The epithelium provides a physical barrier against pathogens. The epithelial cells release cytokines that enhance the inflammatory response and stimulate the production of IgE [16, 24, 27].

The mucus secreted by goblet cells and seromucous glands humidifies air and neutralizes microbes through the IgA and the lysozime present in it. The coordinate ciliary stroke drains mucus and foreign particles from the nasal passage and purifies the inspired air [23, 24].

The cavernous sinuses in the lamina propria regulate the temperature of the inspired air and contribute to the inflammatory response [24].

Because of these important functions, many authors advocate the preservation of the mucosal tissue during turbinate surgery in order to save the nasal physiology and to avoid the adverse effects of a radical approach, like the empty nose syndrome. Therefore, conservative techniques are preferred such as laser surgery, radiofrequency electrocautery, cryosurgery, argon plasma coagulation, ultrasound, traditional or motorized submucosal resection [11, 15, 27].These procedures consist in a reduction of the inflamed erectile tissue with scar formation while preserving the mucosal lining [1].On the contrary, ultrastructural studies demonstrate that these methods produce irreversible changes in the nasal mucosa [28]. Laser treatment resulted in a permanent damage of the mucosal function [29]. Wexler et al. [5] found reduced thickness of the epithelial lining, a marked diminution of venous sinusoids and almost total absence of seromucous glands in the laser-treated areas with compensatory increase of secretory activity in the adjacent regions [5, 11, 18]. Thermal techniques cause coagulation of venous sinuses resulting in fibrosis and scarring of the submucosal tissue. Gindros et al. [7] found loss of cilia after submucous diathermy. Ultrastructural changes after radiofrequency include squamous metaplastic epithelium with basal cells and lack of ciliated, brush cells and columnar cells, fibrosis of the lamina propria, intense inflammatory infiltration and reduction of seromucous glands [7, 19]. It was demonstrated also that thermal techniques cause nerve fibers devitalization resulting in reduced sensation of nasal airflow. Salzano et al. [28] used monofilament test to observe nasal sensitivity before and after radiofrequency, high-frequency, electrocautery and partial inferior turbinotomy. He found that hot techniques had a higher pressure threshold because they caused nervous nasal fibers damage. On the contrary, partial inferior turbinotomy preserved the nasal innervation and the patients treated with this technique required a lower stimulus to produce a touch response [28].

It is our opinion that the partial removal of an altered, damaged mucosa could retain the inflammatory activity in the turbinate and prevent the nasal physiology to be restored. The epithelium of an enlarged turbinate is not able to act as a physical barrier to external agents because of the epithelial shedding, the interruption of intercellular junctions and the lack of goblet cells and cilia. As a result, the submucosal cavernous tissue may continue to undergo swelling through vasodilation, edema, mucous gland stimulation and exudate [27]. This may explain the failure of the mucosal sparing techniques in the long term.

Furthermore, it has been proved that the preservation of the basal cells at the edges of the wound results in migration and proliferation of the epithelial cells in order to repair the injury [30]. It has been already reported that p63 gene plays a central role in the epithelial stem cell self-renewal [31] although critical information on the properties of nasal epithelial stem cells is lacking.

However, the healing process involves other factors including extracellular matrix proteins, like metalloproteinase, laminins, integrins, fibronectins and cytokines [24].

In the present work we examined for the first time the ultrastructural aspects of the nasal mucosa after Microdebrider-assisted turbinoplasty.

Electron Microscopy is largely employed in medicine for the study of healthy and diseased tissues. The Scanning Electron Microscope provides tridimensional detailed images of the cell surface, while Transmission Electron Microscope investigates the fine ultrastructural details in intracellular and extracellular space. Scanning Electron Microscope and Transmission Electron Microscope are valuable tools for a better understanding of the pathogenesis of chronic rhinopathy and the effects of treatment [22, 32].

Our results obtained in preoperative specimens displayed degenerating epithelial cells, with loss of cilia and interruption of tight junctions, gap junctions and desmosomes. The basement membrane was focally thickened, in particular where the epithelial lining was missing, probably to compensate the absence of a physical barrier and as a result of chronic inflammatory state. The stromal tissue was characterized by abundant amorphic substance made of water and proteins and marked inflammatory cells infiltrate, hypertrophic and atrophic glands and engorged thin-walled venous sinuses with abnormal epithelial cells.

An interesting finding was the abundance of fragmented collagen fibers in the sub-epithelial space. We hypothesized that the enlargement of the hypertrophic turbinate exceeds the tensile strength of the extracellular matrix and the cell cytoskeleton resulting in destruction of the network of fibrous proteins and glycosaminoglycans. This may explain the epithelial shedding and the interruption of the intercellular connections. Many authors emphasized the importance of achieving fibrosis in the sub-mucosal tissue in order to prevent the recurrence of turbinate swelling [5, 27, 33]. We believe that scar tissue is not compatible with the physiological characteristics of a healthy mucosa. In contrast, we recommend the preservation of the resection margins to allow the proliferation and migration of the basal cells.

In turn, electron microscopy analysis of postoperative samples revealed a complete re-epithelisation of nasal mucosa with well differentiated columnar ciliated epithelium with the characteristics of the normal mucosa described in literature [22, 24–26, 34, 35] and detected in healthy controls. The amorphic substance decreased and the fibrosis was substituted by bundles of fibrillar collagen. These findings are consistent with the observations obtained with scanning electron microscopy, already reported in our previous study in which the restored epithelial surface showed mucin filaments and normal cilia measuring 24 µm in length and 3 µm in diameter [20].

In the present work we examined the ultrastructural aspects of the nasal mucosa after debridement of the turbinate. The microdebrider precisely removes the soft tissue and the nasal mucosa without burning the resection margins allowing the re-epithelization process. Objective and subjective assessments showed a functional improvement. The patients experienced a relief of symptoms such as nasal obstruction, sneezing, headache, rhinorrhea and the level of satisfaction was high. The visual analogue scale rate decreased with microdebrider after a week. This finding demonstrates improvement of symptoms. The microdebrider resulted more effective than radiofrequency in relieving nasal obstruction. Previously, others reported similar degree of satisfaction and a superior improvement with respect to the laser surgery [8].

The videoendoscopy evaluation showed a great enlargement of the nasal airway in all patients, starting from the first week after the operation. After 4 months the healing process was complete: the nasal mucosa appeared healthy, rose, without sinechiae and crusts. At 4 years after surgery no recidivism was observed and the nasal patency was preserved. We are in agreement with Wexler et al. [5] who support the importance of preserving the general form of the nasal concha to allow more physiologic airflow distribution in the nasal cavity.

The improvement of mucociliary clearance rate confirmed the functional healing of the epithelium and the restoration of the mucociliary system. On the contrary, minimally invasive techniques using thermal energy such as radiofrequency, diathermocoagulation and electrocautery result in loss of cilia due to the reduction of the epithelial perfusion. In fact Salzano et al. [28] found that the mucociliary transport time increased after thermal techniques [28].

After surgery, the rhinomanometric value decreased to 0.11 Pa/cm^3^/s after decongestion reveals that the nasal mucosa retain the physiological vasoreactivity which is important for nasal cycle. The rhinomanometric measurements at 4 years after surgery demonstrated the preservation of physiological nasal resistance in the long term.

## Conclusions

In conclusion, turbinectomy is one of the most performed procedures in rhinological practice and it generally includes the sparing the nasal mucosa, that is the site of important physiologic processes. We confirmed, with the present work, that the total removal of nasal mucosa produces a rapid morphologic and ultrastructural recovery with clinical improvement and satisfaction of the patients.
